# Surgical Repair of a Partial Penile Amputation From a Hair Tourniquet Under Local Anesthesia

**DOI:** 10.7759/cureus.96373

**Published:** 2025-11-08

**Authors:** Eric Midenberg, Uzoma A Anele

**Affiliations:** 1 Urology, University of Louisville School of Medicine, Louisville, USA

**Keywords:** local anesthesia, penile hair tourniquet, penile reconstruction, rare clinical presentation, urethroplasty

## Abstract

Penile hair tourniquet syndrome is a rare but serious condition necessitating prompt diagnosis. It is defined as a circumferential strangulation of the penis secondary to a hair coil and is predominantly encountered in the pediatric population and seldom in adults. The severity of presentation is governed by the duration of hair strangulation, with prolonged cases often necessitating surgical reconstruction. This case report describes the surgical approach in managing a near-complete penile amputation secondary to a penile hair tourniquet. Due to the patient’s comorbidities, the reconstruction was performed under local anesthesia, adding further complexity to this already unique case. At his three-month postoperative appointment, the patient endorsed satisfactory functional outcomes with an excellent cosmetic result. To our knowledge, this is the first report of a surgical reconstruction of a chronic near-complete penile amputation due to a hair tourniquet entirely under local anesthesia.

## Introduction

Penile hair tourniquet syndrome (PHTS) is a rare condition traditionally seen in infants and children, with very few reported cases involving adults [1-5]. PHTS is characterized by circumferential entrapment of a hair fiber or thread around the penis, most commonly occurring proximal to the coronal sulcus [1,2,5]. Early changes arise from lymphatic and venous backflow, resulting in erythema and edema of the glans. This process is followed by strangulation and inhibited delivery of arterial blood supply, leading to distal tissue ischemia and necrosis [2,4-6]. Therefore, early detection and removal of the hair tourniquet becomes crucial in preventing further morbidity. The presentation of prolonged PHTS is variable, with reported complications including urethral transection, urethrocutaneous fistula, penile gangrene, and penile amputation [1,2,5,6]. Therefore, detrimental effects secondary to PHTS pose a unique challenge for the reconstructive urologist. Here, we present a case involving a gentleman with nearly complete penile amputation from a hair tourniquet with additional obstacles for his surgical reconstruction arising from his comorbidities.

## Case presentation

**Figure 1:**
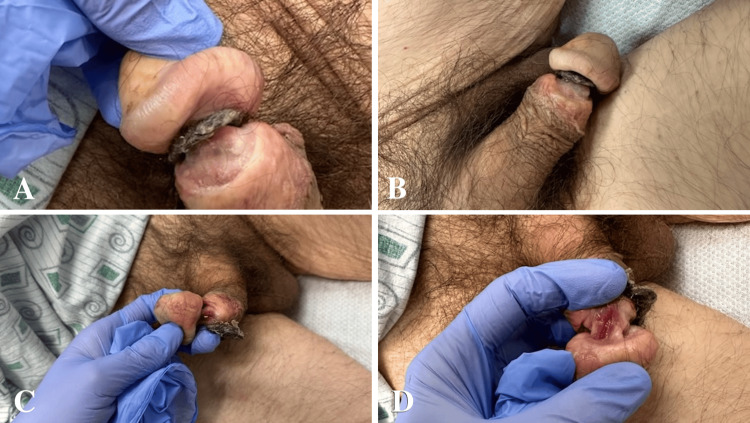
Penile hair tourniquet discovered and removed upon initial hospital presentation (A, B) with consequent erosion of the bilateral corporal cavernosa (C) and partial transection of the ventral urethra (D).

A 48-year-old male with extensive past medical history significant for hypertension, diastolic heart failure, severe chronic obstructive pulmonary disease (COPD) (on 4 L home oxygen), human immunodeficiency virus, and poor functional status necessitating residence at a rehabilitation facility was referred to the urology clinic for evaluation of a penile injury secondary to a hair tourniquet. He initially presented to an outside hospital for acute respiratory failure, at which time a penile hair tourniquet was discovered and released following urologic consultation for difficult catheter placement (Figure 1).

**Figure 2:**
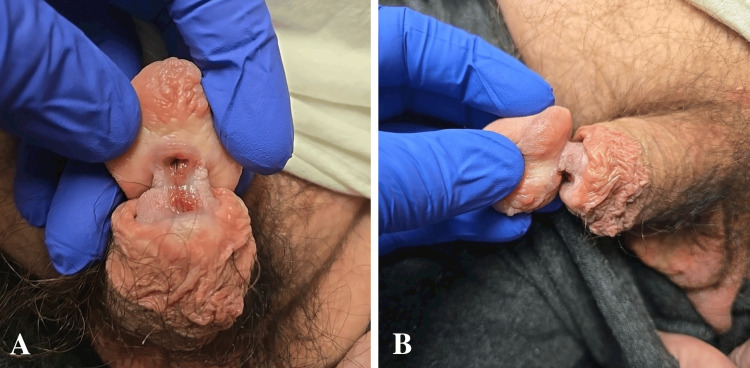
Preoperative in-office physical exam demonstrating near complete transection of the ventral urethra (A) with continuation of the penis maintained by the dorsal aspect of epithelialized spongiosum (B).

At the time of office evaluation, the patient estimated that the penile hair tourniquet injury had been present for approximately one year. Physical examination revealed a circumcised phallus with a chronic-appearing, nearly complete circumferential transection at the level of the distal penile shaft just proximal to the coronal sulcus. The continuity of the penis was maintained by the dorsal aspect of epithelialized spongiosum, resulting in instability of the glans. Additionally, there was complete luminal exposure of the ventral urethra (Figure 2).

Interestingly, he reported no bothersome difficulty or issue achieving satisfactory erections. After discussion of risks and benefits, the patient elected to undergo penile reconstruction.

On the day of surgery, the anesthesia team determined that the patient was at prohibitive risk for general anesthesia or even conscious sedation due to his severe diastolic heart failure and advanced COPD. Given the prospect of canceling the operation, we proposed performing the procedure entirely under local anesthesia, to which the patient consented and elected to proceed.

**Figure 3:**
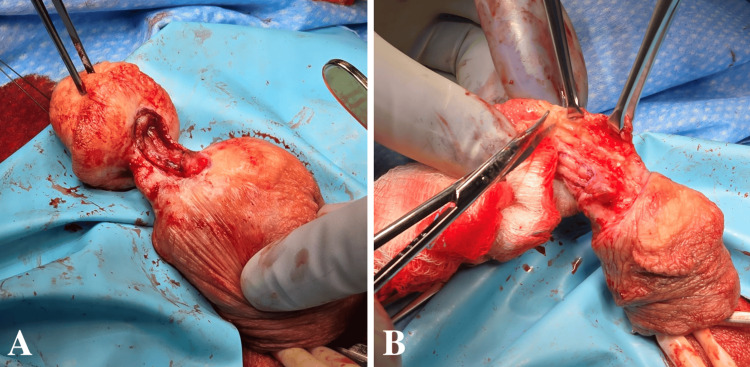
Excision of epithelialized tissues surrounding the corpus spongiosum of the ventral urethra (A) and the distal penile shaft (B).

To begin, a dorsal and penile ring block was performed with 30 mL of 0.25% bupivacaine combined with 4 mg of dexamethasone. A penile tourniquet was applied using a ¼ inch Penrose drain wrapped at the base of the penile shaft. The densely adherent and epithelialized amputated surfaces were systematically and sharply excised beginning with the spongiosum (Figure 3).

**Figure 4:**
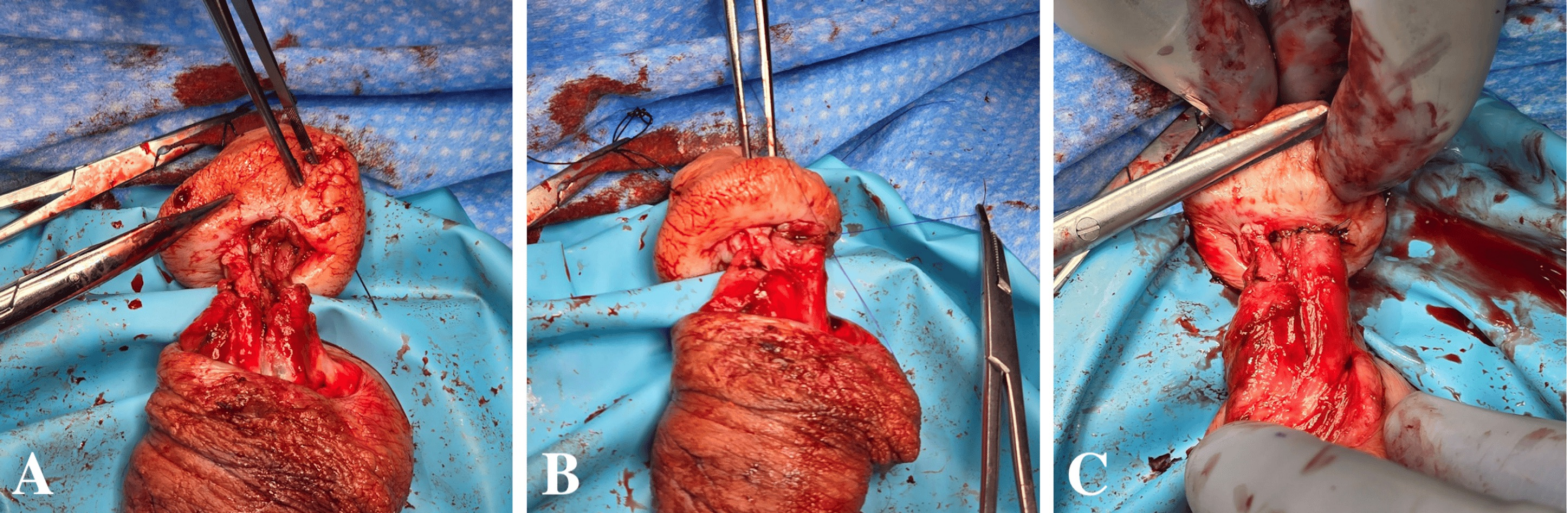
Mobilization of the distal and proximal aspects of the ventral urethral defect (A) with subsequent primary repair (B, C).

The urethra was mobilized, re-aligned, and found to primarily approximate readily without tension. The urethra was calibrated proximally and distally via the defect using a 26Fr Bougie and a Boule dilator to ensure adequate patency. The urethra was approximated using 5-0 polydioxanone suture (PDS) in an interrupted fashion in the midline and then in a running fashion medially to laterally on both sides (Figure 4).

An imbricating layer involving the adjacent Buck’s fascial tissues was then closed over the suture line using 5-0 PDS suture in a running fashion. The repair was confirmed watertight, and the urethral anastomosis was able to easily accommodate a 22Fr Bougie a Boule dilator via the meatus. 

**Figure 5:**
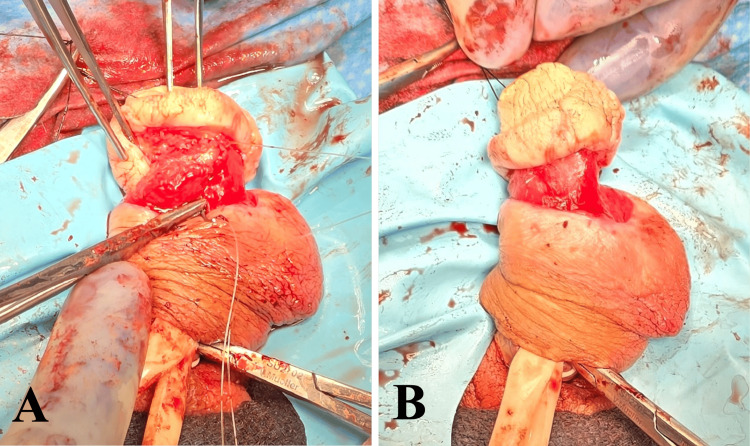
Approximation of corpora to the deep tissues within the glans (A) followed by interposition of dartos flap over the repair (B).

To address the corpora cavernosum, the epithelialized layer was sharply excised from the amputated surfaces. Buck’s fascia overlying the urethra and the lateral tissues were approximated using 4-0 polyglactin 910 suture in a running fashion on both sides. The corpora were then approximated to the deep tissues within the glans using 3-0 PDS in a running fashion on both sides (Figure 5A). A dartos flap was then mobilized from the proximal penile skin and approximated to similar tissue at the level of the inner glans using 3-0 polyglactin 910 suture in a running fashion (Figure 5B).

**Figure 6:**
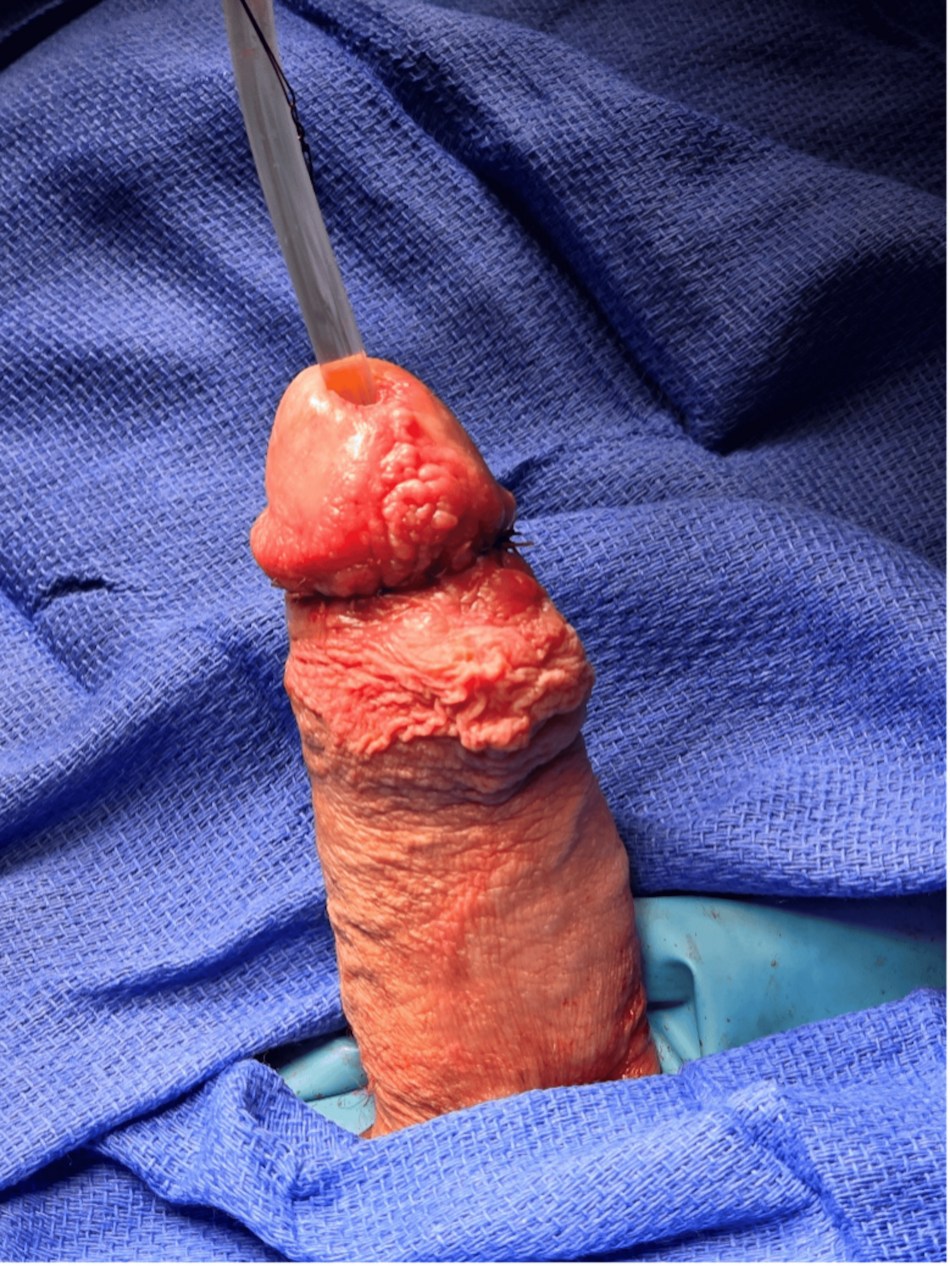
Approximation of skin edges to achieve wound closure.

The skin was then approximated using 3-0 chromic suture in a simple interrupted fashion. The penile tourniquet was removed, and immediate perfusion was appreciated (Figure 6).

An 18Fr Foley catheter was inserted. An additional dorsal and penile block was administered. A bio-occlusive dressing was placed, followed by a temporary self-adherent elastic wrap.

The patient was notably conversant and engaged throughout the procedure with effective analgesia. During the urethral repair, there was a moment of brief focal sensation, which was quickly resolved with the administration of additional local anesthetic.

**Figure 7:**
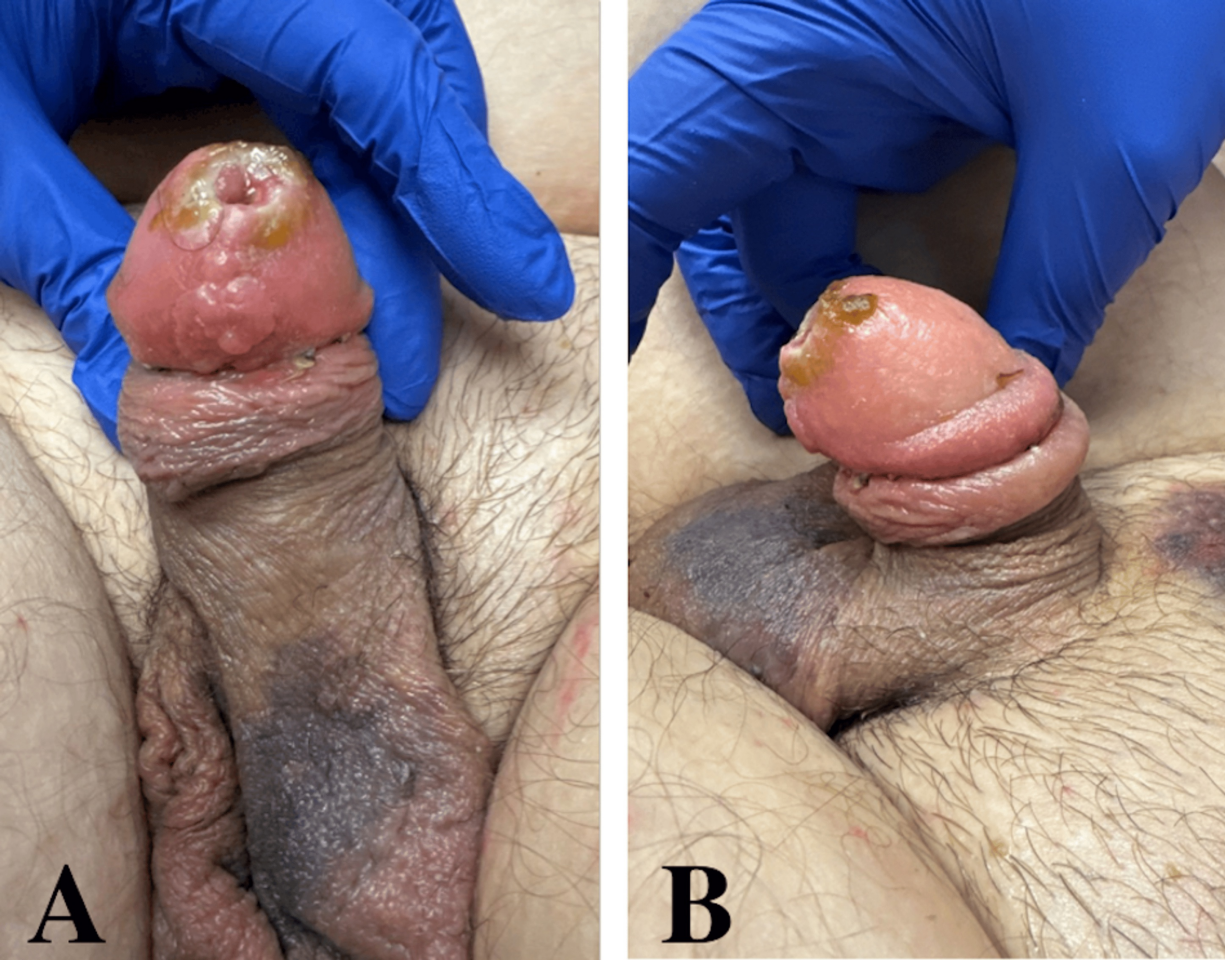
Mild edema and ecchymosis noted in office two weeks postoperatively during removal of the urethral catheter (A, B).

The patient was evaluated in the clinic two weeks postoperatively for catheter removal. The glans appeared viable, and the repair was intact with appropriate signs of healing and mild penile edema (Figure 7).

**Figure 8:**
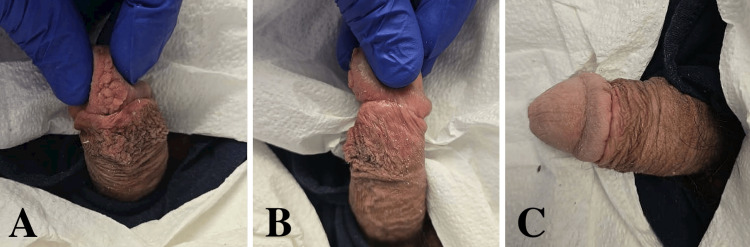
Resolution of wound edema and ecchymosis with appropriate cosmesis noted in office six weeks postoperatively (A-C).

The Foley catheter was removed, and he passed a trial of void. At six weeks postoperatively, uroflowmetry was performed and revealed an inadequate voided volume of 90mL, resulting in a suboptimal assessment of flow dynamics (Qmax = 8.1 mL/s). The post-void residual was minimal at 3 mL, and the patient reported voiding comfortably without difficulty. The penile edema notably resolved, and examination demonstrated restoration of normal penile anatomy (Figure 8).

The patient confirmed maintenance of his baseline sexual functionality.

## Discussion

PHTS is a unique and potentially devastating condition almost exclusively seen in the pediatric population [1-5]. While there are many reported cases of penile strangulation from other foreign objects, there is a paucity of literature detailing the management of severe penile injuries secondary to PHTS in the adult population. Early detection can be challenging as visualization of the hair tourniquet may be difficult due to surrounding edema, or the presentation may mimic benign conditions such as paraphimosis [4,6]. Furthermore, varying knowledge of this condition among medical providers may delay diagnosis. Irrespective of age, it is imperative for the physician to have PHTS on the differential diagnosis when patients present with glandular edema. 

Previous studies involving pediatric patients have detailed the presentation and management of PHTS. Prior circumcision has been proposed as a potential risk factor, as the lack of preputial skin promotes hair tourniquet formation proximal to the exposed coronal sulcus [3]. Urethral injury is a frequently reported finding in advanced cases. A multicenter study performed by Badawy et al. evaluated 25 pediatric patients with severe penile injuries secondary to PHTS, with partial or complete urethral transection noted in 100% of patients [1]. Acini et al. published their experience with PHTS in a small case series of seven children, all of whom sustained partial urethral transection [5]. These data suggest that the dorsal neurovascular bundle is more resilient, as the urethra is often compromised earlier in the ischemic process [5]. Similarly, our patient presented with a partial transection of the urethra. When evaluating patients with PHTS, investigation into urethral injury may be warranted in cases where strangulation has been prolonged and the exam is ambiguous.

Due to the rarity of PHTS, its surgical management is not clearly delineated. The basis of surgical reconstruction in advanced cases should emphasize excision of epithelialized tissues, adequate mobilization of affected structures, followed by urethroplasty with re-approximation of the corporal bodies to the glans to restore orthotopic anatomy with minimal tension [1]. The majority of reported case series favor a single-stage over a multi-stage repair; however, this decision may be influenced by the degree of corporal cavernosal damage [1]. A multi-stage approach may be favored in cases where the glandular blood supply is questionable, which may be indicated by poor glans capillary refill or if continuity of the penis is maintained by a thin pedicle. In our case, the edges of the urethral lumen required minimal debridement, and the glans showed no signs of ischemia, supporting our decision for a single-stage reconstruction. The nature of this injury implies disruption of the dorsal artery and its associated neurovascular bundle. We suspect that perfusion to the glans was preserved through collateral branches of the bulbourethral artery coursing within the residual, incompletely transected corpus spongiosum. Furthermore, previous case series identify urethrocutaneous fistula and urethral stricture to be the most frequent postoperative complications [1,5]. These findings underscore the importance of utilizing interposing dartos fascia over the urethral anastomosis to reduce the risk of future sequelae. 

The feasibility of performing minor penoscrotal surgery under local anesthesia has been well established in the literature [7]. These operations are reliant on their short duration and the adequate pain control achieved with local nerve blocks. To our knowledge, this is the first case report to describe performing urethroplasty with penile reconstruction exclusively under local anesthesia. Our patient was conversant throughout the surgery and endorsed minimal to no discomfort. Maintaining intraoperative nerve blockade through supplementation of local anesthesia when needed may be an alternative option for patients with limited distal anterior urethral stricture disease who are not candidates for general anesthesia or sedation.

## Conclusions

This case report details the surgical management of a 48-year-old male who presented with a nearly complete penile amputation consequent to a chronic penile hair tourniquet. Prolongation of tissue ischemia from a strangulating hair tourniquet may lead to potentially catastrophic sequelae that often warrant surgical reconstruction, emphasizing the importance of prompt diagnosis and treatment.

The patient elected to pursue penile reconstruction. Due to his severe cardiac and pulmonary comorbidities, we performed a single-stage repair entirely under local anesthesia. The surgery was well tolerated with optimal local nerve blockade. Postoperatively, the patient had an excellent functional and cosmetic outcome.
